# Amphotericin B biosynthesis in *Streptomyces nodosus*: quantitative analysis of metabolism via LC–MS/MS based metabolomics for rational design

**DOI:** 10.1186/s12934-020-1290-y

**Published:** 2020-01-31

**Authors:** Bo Zhang, Yi-Teng Zhou, Sheng-Xian Jiang, Yu-Han Zhang, Kai Huang, Zhi-Qiang Liu, Yu-Guo Zheng

**Affiliations:** 1 Key Laboratory of Bioorganic Synthesis of Zhejiang Province, College of Biotechnology and Bioengineering, Zhejiang University of Technology, Hangzhou, 310014 People’s Republic of China; 2 Engineering Research Center of Bioconversion and Bio-purification, Ministry of Education, Zhejiang University of Technology, Hangzhou, 310014 People’s Republic of China

**Keywords:** Amphotericin B, Metabolomics, *Streptomyces nodosus*, Overexpression

## Abstract

**Background:**

Amphotericin B (AmB) is widely used against fungal infection and produced mainly by *Streptomyces nodosus*. Various intracellular metabolites of *S. nodosus* were identified during AmB fermentation, and the key compounds that related to the cell growth and biosynthesis of AmB were analyzed by principal component analysis (PCA) and partial least squares (PLS).

**Results:**

Rational design that based on the results of metabolomics was employed to improve the AmB productivity of *Streptomyces nodosus*, including the overexpression of genes involved in oxygen-taking, precursor-acquiring and product-exporting. The AmB yield of modified strain *S. nodosus* VMR4A was 6.58 g/L, which was increased significantly in comparison with that of strain *S. nodosus* ZJB2016050 (5.16 g/L). This was the highest yield of AmB reported so far, and meanwhile, the amount of by-product amphotericin A (AmA) was decreased by 45%. Moreover, the fermentation time of strain *S. nodosus* VMR4A was shortened by 24 h compared with that of strain. The results indicated that strain *S. nodosus* VMR4A was an excellent candidate for the industrial production of AmB because of its high production yield, low by-product content and the fast cell growth.

**Conclusions:**

This study would lay the foundation for improving the AmB productivity through metabolomics analysis and overexpression of key enzymes.
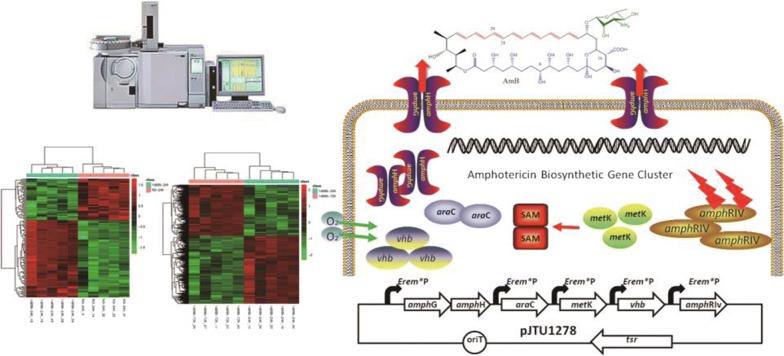

## Background

Amphotericin B (AmB) is a widely-used and irreplaceable therapeutic agent against systematic mycoses with an advantage that its antibiotic resistance is slow to emerge [1]. Activity of AmB against enveloped viruses and pathogenic prion proteins has also been reported [2]. For over 50 years, AmB has been applied in clinical practice and it is still the preferred drug for deep fungal infection nowadays.

**Fig. 1:**
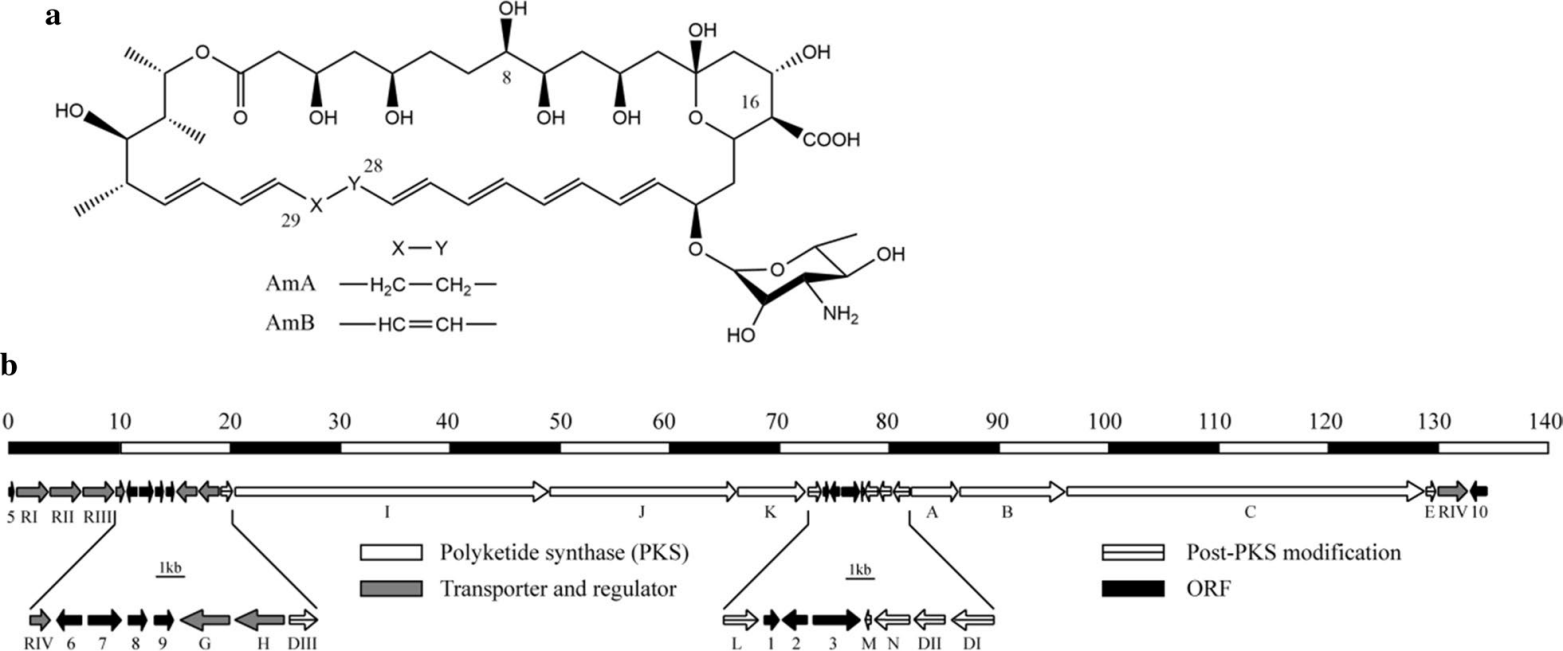
Structures and the antibiotic biosynthesis gene cluster of amphotericin. **a** Structures of amphotericin B and amphotericin A, which are different in the reduction of C28–C29 double bond. **b** The amphotericin biosynthesis gene cluster is organized with PKS genes, post-PKS modification genes, transporter and regulator genes and other ORF genes, which are described by white arrow, white arrow with line, grey arrow and black arrow, respectively

The main AmB-producing microorganism is *Streptomyces nodosus*. Another fungus *Penicillium nalgiovense* Laxa was reported to synthesize AmB recently [3]. However, the low productivity of AmB significantly limits its industrial production and increase the production cost. In pursuit of rational genetic engineering strategies applied on *S. nodosus* to increase its AmB production and novel AmB derivatives, the genome of *S. nodosus* was sequenced [4]. The 7.7 Mb genomic DNA of *S. nodosus* contains 24 biosynthetic gene clusters of polyketides, peptides and terpenes. Amphotericin biosynthetic gene cluster is 135 kb in length and is consisted of polyketide synthase (PKS) genes, post-PKS modifications genes, transporter genes, regulator genes and open reading frame (ORF) genes (Fig. 1). Through reprogramming polyketide synthase and engineering enzymes required for modification of the macrolactone core, some new analogs with different activities have been biosynthesized in *S. nodosus* [5, 6]. However, further metabolic engineering for AmB biosynthesis improvement and industrialization was hindered by complicated regulation mechanism and ambiguous gene function. Therefore, a comprehensive understanding of AmB biosynthesis mechanism in *S. nodosus* at a systematic level is of upper most priority.

With the assistance of principal component analysis (PCA) and partial least squares (PLS) combined with metabolomics, metabolism characteristics of FK506 in *Streptomyces tsukubaensis* [7] and fumaric acid in *Rhizopus oryzae* have been revealed systematically [8]. Metabolomics is also an efficient tool to monitor the type and quantity of intracellular metabolites accurately during fermentation, which was vital for metabolic modification [9]. The key metabolites obtained by PCA, PLS and metabolomics would further guide the metabolic engineering in complicated metabolic network. Hence, we consider metabolomics as an effective approach to reveal the mechanism of AmB production and provide us useful information for metabolic target modifications in *S. nodosus*.

It was reported that the AmB production in *S. nodosus* was improved by expression enhancement (acyl CoA carboxylases, methylmalonyl CoA mutase and phosphopantetheine transferases), glycosylation engineering, optimization of fermentation conditions and modification of amphotericin PKS genes [10, 11]. In our previous study [12], a mutated strain of *S. nodosus* with a high AmB yield was obtained by ultraviolet-nitrosoguanidine (UV-NTG) mutation and significant factors involved in AmB fermentation were also investigated. To further increase the yield of AmB, comprehensive metabolomics analysis of strain *S. nodosus* ZJB2016050 was carried out for the first time in this study to identify the distribution and relative quality of metabolites and 8 pathways that associated with AmB biosynthesis. Within this study, a combinatorial overexpression of key genes that selected on the basis of metabolomics results, experimental verification and previous report for secondary metabolites synthesis were conducted. The engineered strain constructed in this study showed high AmB yield and low by-product yield, indicating its potential in large-scale production.

## Results

### Cell growth and metabolite patterns for AmB production

**Fig. 2:**
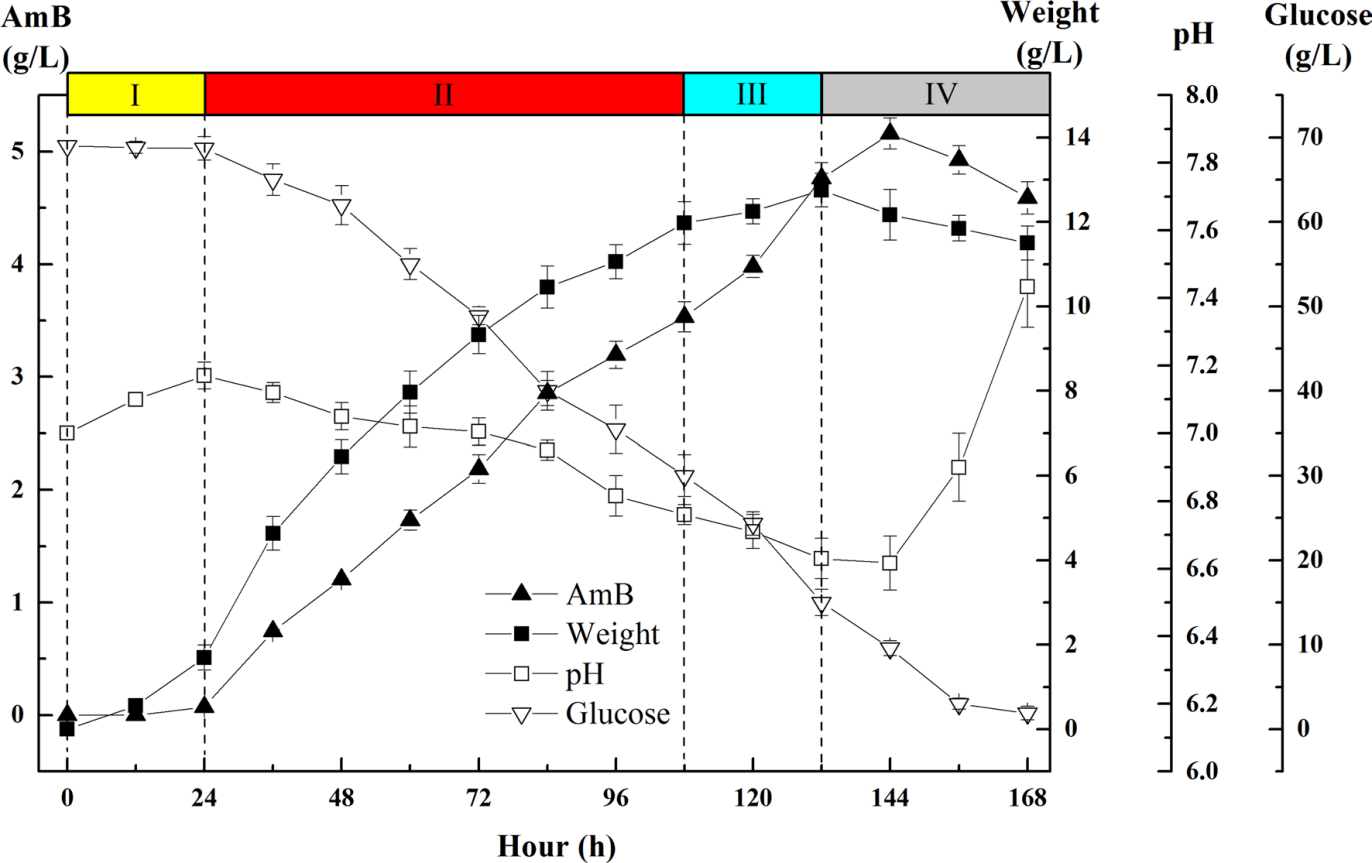
Fermentation profiles for strain *S. nodosus* ZJB2016050. Four profiles are illustrated in the line chart, including the yield of AmB, dry cell weight, pH and residual glucose. The whole process could be divided into four phases, lag phase (0–24 h), exponential phase (24–108 h), stationary phase (108–132 h) and decline phase (132–168 h). Each value is a mean of three experiments. Error bars show standard derivation among three experiments

As shown in Fig. 2, the fermentation process of strain *S. nodosus* ZJB2016050 could be divided into four phases, I (0–24 h), II (24–108 h), III (108–132 h) and IV (132–144 h). The dry cell weight (DCW) increased to 1.69 g/L during lag phase (I) and to 12.75 g/L during the exponential phase (II). During the stationary phase (III), the cell biomass reached to 12.87 g/L and then stopped accumulating. A significant increase was observed in AmB production with a maximum yield of 5.16 g/L and the DCW decreased to 12.17 g/L at 144 h. In AmB-synthesized phase (IV), the pH value decreased from 7.0 to 6.6 with a glucose consumption rate of 0.51 g/h. After the maximum yield of AmB was reached, pH began to increase from pH 6.6 to pH 7.4 within 24 h and the biomass of *S. nodosus* ZJB2016050 also decreased during this phase. According to the results above, strain *S. nodosus* ZJB2016050 exhibited different fermentation characteristics during the fermentation time course. However, it was difficult to find the bottlenecks that restricted the AmB production only on the basis of fermentation results. Therefore, multivariate statistical analysis (PCA and PLS-DA) were performed based on a great quantity of data collected from LC–MS/MS with an intension to rationally improve the AmB production.

### LC–MS/MS based metabolomics for AmB biosynthesis

Significantly discrepant metabolites were identified among four groups with q < 0.05 and fold change > 1.20 or < 0.83. Results indicated that 11,145 differential ions (positive mode) and 3068 differential ions (negative mode) were identified within group 24 h and group 72 h. Between group 72 h and group 120 h, there were 4968 differential ions (positive mode) and 890 differential ions (negative mode). There were 3136 differential ions (positive mode) and 1893 differential ions (negative mode) between group 120 h and group 156 h (Additional file 1: Table S3).

Analysis of the metabolic pathways and assumed structure of the metabolites were facilitated with KEGG database. In four sample groups, 7763 ions of level 1 ions and 4575 ions of level 2 ions were identified (positive mode), meanwhile, 2460 ions in level 1 and 1398 ions in 2 were obtained (negative mode). The extra information of the differential ions was listed in Additional file 1: Table S3.

**Fig. 3:**
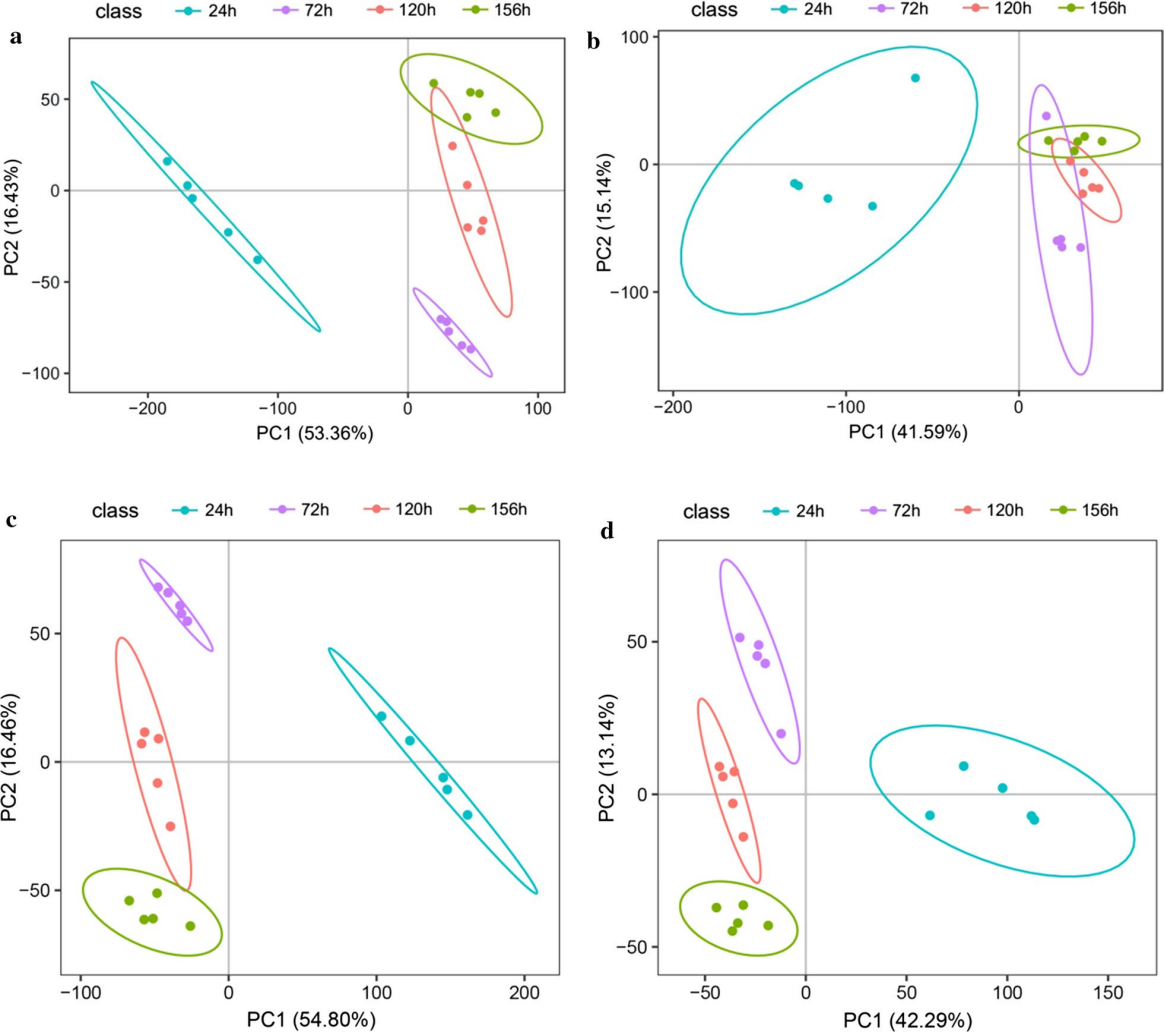
PCA and PLS-DA analysis of intracellular metabolites at different fermentation time points. The samples were withdrawn from the cultivation at 24, 72, 120 and 156 h. **a** PCA scores scatter plot in positive ion scan modes. **b** PCA scores scatter plot negative ion scan modes. **c** PLS-DA scores scatter plot in positive ion scan modes. **d** PLS-DA scores scatter plot in negative ion scan modes. In order to assess the accuracy and stability of the equipment state during detection and collection process, the quality control samples (the mixture of all samples) were prepared in advance, and then were carried out every 10 samples

PCA and PLS-DA were performed to test the relativity of various metabolites and AmB yield. PCA plots including QC samples were provided to evaluate the data quality (Additional file 1: Fig. S5). The PCA scores plot indicated four clusters from data obtained in different fermentation time points (24, 72, 120 and 156 h) of *S*. *nodosus* (Fig. 3a, b). Samples from group 24 h exhibited a slack mode different from samples in other groups. Group 120 h and group 156 h were much closer to each other than the distance between group 24 h and group 120 h or group 156 h. For the obvious discrepancy among the groups, PLS-DA analysis was carried out for further investigation of the differences during the whole fermentation process of *S. nodosus* (Fig. 3c, d).

### Systematic analysis of metabolites associated with AmB biosynthesis

**Fig. 4:**
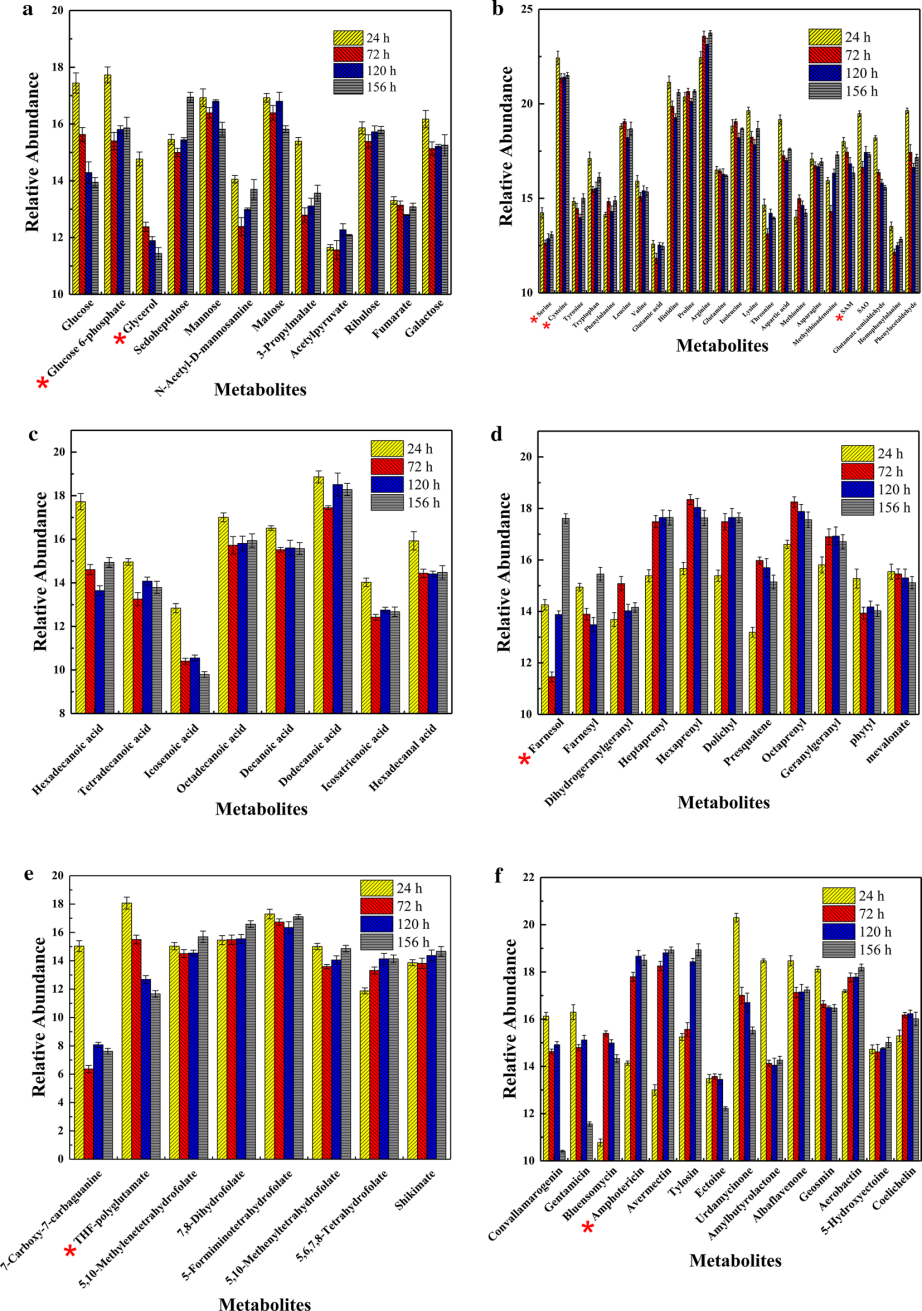
The relative abundances of various intracellular metabolites in different fermentation period. Metabolites were analyzed to investigate the differences during the whole fermentation process of *S. nodosus*, including metabolism of amino acid, sugar, fatty acid, terpenoid backbone, folate biosynthesis and the other secondary metabolites. **a** Amino acid metabolism, **b** sugar metabolism and central metabolic pathway, **c** fatty acid biosynthesis, **d** terpenoid backbone biosynthesis, **e** folate biosynthesis and one carbon pool by folate, **f** secondary metabolites and antibiotics. Metabolites annotation have been checked by authentic standards (Glucose 6-phosphate, Glycerol, serine, cysteine, SAM, Farnesol, THF-polyglutamate, Amphotericin), Red * indicates authentic standards. The error bars represent standard deviations of five values

Metabolites were analyzed to investigate the differences during the whole fermentation process of *S. nodosus*, including the metabolism of amino acids, sugar, fatty acids, terpenoid backbone, folate biosynthesis and other secondary metabolites. The key pathways and related metabolites were summarized in Additional file 1: Table S4. As shown in Fig. 4a, type 1 included sugar metabolism and central metabolic pathway (Fig. 4a), which supported the energy metabolism and the biosynthesis of AmB precursors [13]. All intracellular metabolites reduced during the exponential phase of growth, such as glucose, glucose-6-phosphate, fructose-6-phosphate, mannose, and glycerol triphosphate, but varied in the subsequent stages. Mannose (VIP, 1.89), which is the precursor of trehalosamine during the process of AmB biosynthesis, increased from 72 to 120 h and then decreased. Moreover, glucose and 3-phosphate glycerol also showed downtrend in the fermentation process. Regarding to this phenomenon, the addition of glucose and 3-phosphate glycerol during the fermentation process would be one of the strategies to improve AmB production.

Type 2 metabolites involved in amino acid metabolism (Fig. 4b), which not only contributes primarily to the cell growth, but also plays a key roles in the synthesis of secondary metabolites. The majority of the amino acids displayed similar trend during fermentation. Initially, the amino acid content declined from 24 to 72 h, then continuously declined from 72 to 120 h, and finally increased or slightly fluctuated from 120 to 156 h. According to the fermentation parameters, it showed a rapid growth of bacteria and accumulation of AmB from 24 to 120 h and a slow decrease of dry weight and AmB from 120 to 156 h. Amino acids with low variable importance in the projection (VIP), including serine, cysteine, valine, methionine, glutamine and asparagine, exhibited no significant changes in relative quantity or ratio. Other amino acids with high VIP value identified and metabolites related to amino acid metabolism were also listed (Additional file 1: Table S4). Moreover, in methionine and cysteine metabolism, *S*-adenosyl-l-methionine (SAM) with high VIP exhibited a downtrend during the fermentation, indicating a shortage of SAM which is an important component as methyl donor to synthesis of nucleotides and proteins [14]. In this study, only metabolites with VIP > 1 were regarded as statistically significant compounds that contributing the most into groups’ discrimination. To verify the analysis results, 1 mM cysteine, alanine, serine, threonine, arginine, proline and SAM were selected to test the improvement of AmB production in *S. nodosus* respectively. As shown in Additional file 1: Fig. S4, serine, alanine, arginine, proline and SAM increased the biosynthesis of AmB significantly. Interestingly, the production of AmA also increased with high concentration of amino acids.

Type 3 metabolites constituted the fatty acid biosynthesis, which is another important pathway to obtain energy for metabolism and growth (Fig. 4c). Intracellular metabolites, such as hexadecenoic acid, tetradecanoic acid, decanoic acid, dodecanoic acid and icosatrienoic acid, displayed a downtrend during the exponential phase and an accumulation at the latter fermentation phases. To be noticed, when oxygen is sufficient, metabolism of fatty acid can release a large amount of energy and acetyl-CoA, which could be carboxylated to form malonyl-CoA. The basic substances to synthesize polyketide macrolactone rings of AmB are malonyl-CoA and methylmalonyl-CoA [15]. Therefore, oxygen supply is also a crucial factor to provide sufficient precursor during AmB biosynthesis according to the trend of metabolites.

Type 4 metabolites contributed to the terpenoid backbone biosynthesis, which plays a crucial role in metabolism, structure and signal transmission (Fig. 4d). Most relative abundance of metabolites initially increased at the exponential phase, for instance, hexaprenyl, presqualene and octaprenyl, indicating a potential relationship with the primary cell growth and synthesis of secondary metabolites. Subsequently, these metabolites remained stable or slightly decreased with exceptions of farnesol (VIP, 3.45) and farnesyl diphosphate (VIP, 3.13), which decreased dramatically at the exponential phase. Hexaprenyl, presqualene, octaprenylfarnesol and farnesyl diphosphate are the intermediates in both mevalonate and non-mevalonate pathways, which are used for biosynthesis of terpenes, terpenoids, and sterols. The downtrend of these compounds at the exponential phase, which is also the fast AmB synthesis period, indicated the metabolic flux inflow from competitive terpene pathway to AmB synthesis.

Type 5 metabolites were mainly involved in the folate biosynthesis and one carbon pool (Fig. 4e). Identified metabolites of folate biosynthesis showed higher VIPs compared to the metabolites in other pathways. The relative abundance of 7-carboxy-7-carbaguanine, THF-polyglutamate, 5-formiminotetrahydrofolate, 5,10-methylenetetrahydrofolate and 5,10-Methenyltetrahydrofolate decreased first and then slightly increased. Especially, the content of 7-carboxy-7-carbaguanine (VIP, 3.04) reduced by 406-folds. In addition, the content of 5,6,7,8-tetrahydrofolate kept increasing in the whole fermentation process and 7,8-dihydrofolate (VIP, 2.43) remained steady firstly and increased in the last phase. Folate is an important carbon unit involved in the biosynthesis of nucleic acids, amino acids and panoic acid in organisms with another carbon unit, methionine derivative. Type 6 metabolites consisted of the biosynthesis of secondary metabolites and antibiotics (Fig. 4f). Seven identified metabolites, the content trends of which were consistent with the results from AntiSMASH2, which is the software that predicts microbial secondary metabolites. These metabolites were ectoine, aerobactin, albaflavenone, aerobactin, geosmin, urdamycin and butyrolactone. There are 24 different kinds of secondary metabolites clusters in *S. nodosus* identified by AntiSMASH2, and reduction or elimination of their biosynthesis in the fermentation process would not only save carbon source and energy, but also reduce the force of consumption, which could in turn increase the production of AmB. More importantly, metabolites profiling showed accumulation of AmB intracellular which indicated the export process should be modified.

Based on the analysis of the differences in metabolomics profiling, as well as their relationships with amphotericin biosynthesis, two strategies were provided to promote AmB production, including rational addition of key metabolites, which might promote biosynthesis of deficient metabolites and availability of key precursors, and rational expression of key genes, which enhance the biosynthesis of AmB by strain itself. However, too much addition of metabolites increased the production cost of AmB. Hence, we employed metabolic targets modification (Metabolic target: SAM, oxygen acquisition, metabolites transporter, pathway-specific activator) to increase AmB production economically according to three principles: (a) metabolism analysis via LC–MS/MS; (b) experimental verification; (c) previous report for secondary metabolite synthesis.

### AmB production enhancement by rational expression of key genes based on metabolomics *S*-adenosyl-methionine synthase and dimeric hemoglobin

**Fig. 5:**
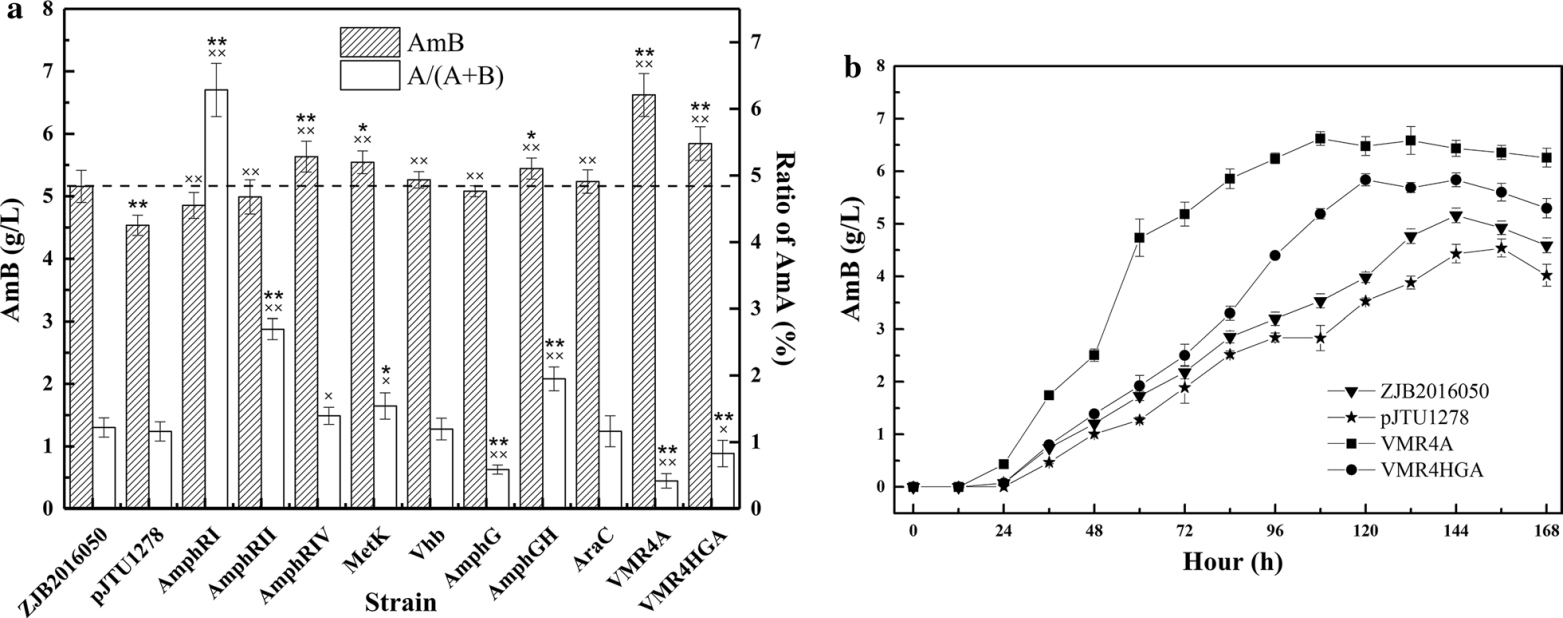
AmB production associated with genes overexpression and the fermentation time courses. **a** AmB production associated with genes overexpression in various engineered strains, the genetically engineered strains wereall constructed from primitive stain, *S. nodosus* ZJB2016050. ZJB2016050 represents strain *S. nodosus* ZJB2016050, pJTU1278 represents strain *S. nodosus* ZJB2016050 with plasmid pJTU1278. vhb, metK, amphRI, amphRIV, amphGH, amphG and araC represent overexpression of gene *vhb*, *metK*, *amphRI*, *amphRIV*, *amphH*, *amphG* and *araC*, respectively, in strain *S. nodosus* ZJB2016050 with plasmid pJTU1278. VMR4A and VMR4HGA were strain overexpressed four genes (*vhb*, *metK*, *amphRIV* and *araC* connected by ermE*p) and six genes (*vhb*, *metK*, *amphRIV*, *amphH*, *amphG* and *araC* connected by ermE*p), respectively. Samples were collected from soluble fermentation at 144 h, and the AmB concentration and ration of AmA were detected and analyzed, respectively. **b** Fermentation time course for strain *S. nodosus* ZJB2016050, pJTU1278, VMR4A and VMR4HGA. The ZJB2016050 and pJTU1278 were primitive strain and the strain with empty vector, respectively. VMR4A and VMR4HGA were strain overexpressed four genes (*vhb*, *metK*, *amphRIV* and *araC* connected by ermE*p) and six genes (*vhb*, *metK*, *amphRIV*, *amphH*, *amphG* and *araC* connected by ermE*p), respectively. Each value is a mean of three experiments. Error bars show standard derivation among three experiments. Symbol ‘*’ means the experimental strain compared with the original strain ZJB2016050 and ^×^ means the experimental strain compare with strain with vector pJTU1278 (*p < 0.05, **p < 0.01, ^×^*p *< 0.05 and ^××^*p *< 0.01)

Recent studies have shown that gene overexpression is an effective strategy to increase antibiotic production [16–18]. However, irrational gene overexpression was barely useful, for instance, *aver* gene was the avermectin pathway-specific regulatory factor and overexpression of *aver* gene was reported to cause the decrease of avermectin production [19]. The same result was reported of SAM biosynthesis gene in novobiocin production [20]. According to the results of metabolic analysis, SAM exhibited a downtrend in the whole fermentation process with high VIP, indicating a shortage of SAM, which is also a crucial component as methyl donor to the synthesis of nucleotides, proteins and activated acyl units. Hence, SAM synthase (coded by *metK* gene, Genbank accession number: AJE39717.1) was overexpressed in some previous studies for its broad function in a variety of synthetic and regulatory reactions which involved in diverse vital movements [21, 22]. In this study, AmB yield increased by 22.1% and reached 5.55 g/L with *metK* gene overexpression in *S. nodosus* (Fig. 5a). SAM is not directly involved in amphotericin biosynthesis, however, the metabolite adding experiments have proved that the supplement of SAM could promote the synthesis of AmB (Additional file 1: Fig. S4), and the precursor (acyl units, malonyl CoA and (2S)-methylmalonyl CoA) supplement for AmB was associated with SAM. Moreover, DNA methylation might affect the expression of biosynthetic genes for amphotericin or other metabolites. Hence, we believe that overexpression of SAM synthase was an efficient strategy to increase the synthesis of AmB.

*Vitreoscilla* hemoglobin (VHb, Genbank accession number: JN418989.1) has been proved to increase the growth and productivity of microorganisms under oxygen-limited conditions. It has been overexpressed in various hosts [23], including bacteria [24, 25], yeast [26] and plants [27]. According to our previous study, the oxygen supply during the fermentation process of *S. nodosus* was insufficient due to the increased viscosity of the fermentation liquid. In Type 3 metabolites, the substances related to fatty acid metabolism decreased obviously. Fatty acids can oxidize and release a large amount of energy and acetylcoenzyme A, then generate carboxylated malonyl coenzyme A and methyl malonyl coenzyme A, which are important precursors for the synthesis of amphotericin. Previous studies have reported the importance of precursors supplement and the overexpression of genes involved in precursors supplement [4]. Hence, *vhb* gene was cloned into plasmid pJTU1278 and controlled under the strong constitutive ermE* promoter [28]. Overexpression of *vhb* gene in strain *S. nodosus* ZJB2016050 promoted the production of AmB by 15.9%, as the results showed in Fig. 5a.

### Pathway-specific regulatory and global regulatory

For secondary metabolites biosynthesis, overexpression of the regulatory factor, *amphRI* and *amphRII* gene, in strain *S. nodosus* ZJB2016050 was conducted but resulted in limited influence on AmB production (Fig. 5a). However, overexpression of another AmB pathway-specific regulatory gene *ampRIV* (Genbank accession number: AJE39070.1) showed a positive effect on AmB production with an AmB-yield improvement to 5.64 g/L (Fig. 5a). Gene *araC* encoded protein is the global regulator of Streptomyces. It belongs to the transcriptional regulatory protein of AraC/XylS family, which involved in a variety of metabolic processes, such as secondary metabolite production, differentiation, carbon utilization, etc., in Streptomyces. O. Sprusanský, et al. proposed that the glyceraldehyde 3-phosphate dehydrogenase (gap) regulator (GapR), a member of the AraC/XylS family of transcriptional activators, might respond to a common product of glucose and glycogen catabolism to activate gap transcription. Glycogen accumulation and degradation may also play a role in morphological differentiation. The Gap-P induction by glucose suggested a physiological role in glycolysis regulation and differentiation [29]. However, Di Sun, et al. demonstrated that a novel AraC-family transcriptional regulator, SAV742, was a global regulator that negatively controlled avermectin biosynthesis and cell growth. Deletion of the corresponding gene, *sav_742*, increased the avermectin production and dry cell weight [30]. It is interesting that *araC* gene showed positive and negative effects for various secondary metabolite production. For the function of carbon utilization that *araC* gene involved in and the down trend of intermediate metabolite in glycolysis and fatty acid metabolism, we selected *araC* as our gene targets. Results showed a positive effect of gene *araC* (GenBank accession number: AJE40807.1) on AmB production, and the yield of AmB increased to 5.24 g/L when *araC* was overexpressed (Fig. 5a).

### ABC transporter

Transport proteins involved in the transport process of antibiotics and their synthetic precursors, which could improve the drug resistance, are of great importance in the synthesis and secretion of antibiotics [31]. Enhancing the transport efficiency of target compounds from intracellular to extracellular is an effective strategy in metabolic engineering for the improvement of antibiotics production. For example, overexpression of *avtAB* gene, which encodes a transport protein, could enhance the production of avermectin in *Streptomyces avermitilis* by twofold [32]. In metabolic profile analysis of *S. nodosus*, the AmB content increased both intracellularly and extracellularly and remained constant at higher level during the fermentation (Figs. 2, 4f). Considering that antibiotics accumulate gradually in the interior of cells, we determined to overexpress the transport genes of AmB. Gene *amphG* (Genbank accession number: AAK73498.1) and *amphH* (Genbank accession number: AAK73499.1) exhibit high identity to ATP-binding transporters, hence, we overexpressed *amphG* and *amphH* in strain *S. nodosus* ZJB2016050 separately and results showed that the AmB-yield increased by 11.8% and 19.8%, respectively. Co-expression of *amphH* and *amphG* in *S. nodosus* ZJB2016050 improved the yield of AmB to 5.44 g/L. To further increase the production of AmB in strain *S. nodosus* ZJB2016050, genes *amphG*, *amphH*, *metK*, *araC*, *vhb*, *amphRIV* were co-expressed with plasmid pJTU1278 (strain *S. nodosus* VMR4A) and each gene was controlled under the ermE* promoter. RT-qPCR analysis of transcriptional level of single gene and multi-genes expression in *S. nodosus* VMR4A showed the different degree of expression for *metK*, *amphRIV*, *amphHG* and *araC* genes (Additional file 1: Fig. S2). Our results indicated that single gene modification did not show remarkable influence on antibiotics production improvement, only combinatorial gene modification improved the production significantly. Fermentation of strain *S. nodosus* VMR4A showed that the yield of AmB increased to 6.58 g/L, with a 28.0% improvement compared to strain *S. nodosus* ZJB2016050, whereas, by-product AmA decreased by 45% and fermentation period shortened for 24 h (Fig. 5b).

## Discussion

In this study, we present a novel approach of strain modification via LC–MS/MS-based metabolomics for the improvement of AmB production, which is more rapid and efficient in comparison with the traditional non-rational method. According to the analysis of the metabolomics results, the contents of metabolites were correlated with biomass accumulation and AmB biosynthesis in *S. nodosus* ZJB2016050. Results revealed significant differences of production capability and fermentation characteristics, reflecting intracellular physiology and metabolism.

The mechanism of AmB biosynthesis and the relationships between AmB synthesis and extracellular carbon source, precursor, pH and residual sugar have been studied previously [4]. In this work, 28 metabolites that associated with the source of precursor or energy metabolism were determined to be crucial for AmB biosynthesis according to the statistical analysis of differential metabolites at 24 h, 72 h, 120 h and 156 h of fermentation process. To discover the significance of relevant metabolic pathways for AmB biosynthesis, pathway enrichment analysis was employed to reveal metabolic mechanism. The differential metabolites were then analyzed through pathway enrichment for their functionin AmB production based on KEGG database. The significances of enriched pathways were evaluated according to their p value as shown in Additional file 1: Fig. S3. The main differential metabolites examined in this study were steroid, ubiquinone, unsaturated fatty acids, drug metabolism-cytochrome P450, tyrosine, tryptophan, lysine, terpenoid backbone, neomycin, kanamycin and gentamicin. There are 24 clusters including PKS genes and non-ribosomal peptide synthase genes in *S. nodosus* and some secondary metabolites showed uptrend during fermentation which is a disadvantage for AmB production. Because the biosynthesis of antibiotic consumes a large amount of resources, knockout or knockdown gene clusters that encode secondary metabolites would save energy and redirect the metabolic flux from other secondary metabolites to AmB formation, which could further increase the accumulation of AmB.

Addition of metabolites based on the metabolomics results is a general and efficient strategy to enhance the biosynthesis of target compound, and it is common in experimental stage [33]. However, the expensive precursor and complicated fed-batch fermentation limited the industrialization of this approach. In this research, results of metabolomics were verified based on metabolites addition, which was useful for medium optimization (Additional file 1: Fig. S4). Moreover, genetic modification was executed according to metabolomics analysis and results of metabolites addition to promote AmB production. We screened the genes that were involved in oxygen-taking, precursor-acquiring and product-exporting. Through overexpression of gene *vhb*, *metK*, *amphRIV*, *amphHG* and *araC*, respectively, the highest yield of AmB increased by 24.1% and the co-overexpression of gene *vhb*, *metK*, *amphRIV* and *araC* promoted the production of AmB to the value of 6.58 g/L in flask shake. It is the highest yield of AmB reported so far with 24 h less of time. Moreover, the accumulation of by-product AmA decreased by 45%. However, the yield of by-product AmA in engineered bacteria was unpredictable, as AmA obviously increased in the strain with *amphRI* or *amphRII* gene overexpressed and decreased in the strain with *amphG* or others genes overexpressed (Fig. 5). Caffrey reported that ER5 domain of *amph*C in amphotericin gene cluster playsa critical role in the biosynthesis of AmA and AmB [2]. It is concluded that the expression of different genes lead to the changes of metabolic flux, which affect the biosynthesis ratio for AmA and AmB. Further metabolomics study between different genotype should be employed to explore the mechanism of AmA biosynthesis,

As is known, the supply of precursors determines the production of end product and by-product. Metabolites acyl CoA and malonyl CoA are involved in the biosynthesis of other metabolites. These crucial metabolites are mainly consumed or accumulated from the amino acid, sugar, fatty acid, terpenoid backbone and folate biosynthesis. In addition, these differential metabolites also reflect key substances in the growth process of bacteria [34, 35]. Recently, rate limiting step for amino acid and fatty acid synthesis has been discovered [36–38]. The improvement of enzyme activity also helps cell precursor accumulation for AmB synthesis. These strategies will further support metabolic engineering for overproduction of AmB.

## Conclusions

In this study, metabolomics profiling combined with rational gene overexpression could provide a simple and common metabolic engineering strategy for the improvement of target compound. We have identified 28 metabolites as key factors and 6 pathways were associated closely with AmB production. Based on these results, six genes were screened and demonstrated to be effective for the biosynthesis of AmB. Compared with the approach of key metabolites addition during the fermentation, overexpression of *vhb*, *metK*, *amphRIV*, *amphHG* and *araC* genes is a more economical and efficient way for large-scale industrial production of AmB. Furthermore, metabolic network model should be employed combining with metabolomics and gene overexpression results for titer promotion.

## Methods

### Strains, media, and growth conditions

All strains and recombinant plasmids with characteristics and resource used in this article are listed in Additional file 1: Table S1, including the obtained mutated strain *Streptomyces nodosus* ZJB2016050 (CCTCC M2017426, China Center for Type Culture Collection, Wuhan, China). Luria-bertai medium and GYM medium were used for *Escherichia coli* and *S. nodosus* cultivation, respectively. Luria-bertai medium (1 L): 5 g yeast extract, 10 g tryptone, 10 g NaCl. GYM agar slants (1 L): 4 g glucose, 10 g malt extract, 4 g yeast extract, 2 g CaCO_3_ and 20 g agar. S1 seed culture (1L): 10 g yeast extract, 10 g glucose, 15 g peptone, 5 g NaCl and 1 g CaCO_3_. F2 fermentation medium (1L): 69 g glucose, 25 g beef extract, 9 g CaCO_3_ and 0.1 g KH_2_PO_4_. All medium were adjusted to pH 7.0 before autoclaving at 115 °C for 30 min, when needed amino acids or other metabolites were added in the medium. Agar slants were incubated at 28 °C for 4–10 days [39]. *S. nodosus* seed culture was cultivated in 50 mL/250 mL shake flask with S1 seed culture at 25 °C for 48 h and transferred to 100 mL/500 mL shake flask with F2 fermentation medium for 4–7 days.

### Plasmid construction

The sequences of ermE*p promoter and *metK* gene were artificially synthesized with a *Xba*I site before ermE*p promoter, a *Hin*dIII site between promoter and *metK* start codon, a *Bam*HI site and a *Kpn*I site following the stop codon and terminator respectively. The synthesized sequence was cloned into plasmid pJTU1278 with the restriction sites *Xba*I and *Kpn*I and named as pJTU-EmetK. Similarly, the *amphRI*, *amphRII*, *amphRIV*, *vhb*, *amphG*, *amphHG* and *araC* were also cloned into plasmid pJTU-EmetK, replacing the sequence between the restriction sites *Hin*dIII and *Bam*HI, named as pJTU-EamphRI, pJTU-EamphRII, pJTU-EamphRIV, pJTU-Evhb, pJTU-EamphG, pJTU-EamphHG and pJTU-EaraC, respectively (see Additional file 1: Fig. S1). Digestion by isocaudamer of *Bam*HI and *Bgl*II, pJTU-VM was then constructed by cloned ermE*p and *metK* between *Bam*HI and *Kpn*I from pJTU-Evhb. pJTU-VMR4A and pJTU-VMR4HGA were constructed from pJTU-VM by One Step Cloning Kit (Vazyme Biotech Co., Ltd, Nanjing, China), which is in-Fusion Cloning and Multi-sequence assembly. Conjugative plasmid transfer was executed as previously described [40]. All used primers are listed in supplementary Additional file 1: Table S2.

### AmB analysis, sampling, quenching, and extraction of intracellular metabolites

In order to analyze the AmB production, 1 mL of fermentation broth and 9 mL of dimethyl sulfoxide were mixed and oscillated for 30 min, and the supernatant was diluted by methanol after centrifugation. AmA and AmB quantification by high performance liquid chromatography (HPLC) was performed on a LDC 3200 analytical system (LDC ANALYTICAL INC., New York, USA), equipped with a Agilent C18 reversed-phase column (5 μm, 4.6 × 150 mm, Agilent Technologies Inc., California, USA) and a UV–vis detector. AmA and AmB were analyzed at 304 nm and 405 nm, respectively. The column was eluted with 20% (v/v) methanol, 35% (v/v) acetonitrile and 45% (v/v) double-distilled water at a flow rate of 1 mL/min. In addition, the commercial standard of AmB was obtained from Sigma-Aldrich (CAS: 1397-89-3).

Samples at different time points of *S. nodosus* ZJB2016050 fermentation, 24 h, 72 h, 120 h and 156 h, were collected and immediately centrifuged at 9000*g* for 3 min. Subsequently, cell pellet was washed with saline solution three times and stored at − 80 °C until use. For intracellular metabolites extraction, 0.1 g samples were taken in 1.5 mL Eppendorf tubes and resuspended with 800 μL of pre-cooled methanol: water (1:1, by volume) solution. Cell samples were then lysed with steel balls in a TissueLyser at 35 HZ for 4 min. The mixture was rested at − 20 °C for 2 h, and then centrifuged at 30,000*g* for 20 min (4 °C). The supernatant was further analyzed for the identification of intracellular metabolites.

### Detection and identification of intracellular metabolites by LC–MS/MS

The intracellular metabolites were detected by LC–MS/MS system with 10 μL of injection volume. The ultra-performance liquid chromatography (UPLC) system (Waters, Milford, USA) was equipped with a ACQUITY UPLC BEH C18 column (100 mm × 2.1 mm, 1.7 μm, Waters, Milford, USA). The column was gradiently eluted with solvent A (H_2_O with 0.1% CHCOOH) and solvent B (CH_3_CN with 0.1% CHCOOH) at a flow rate of 0.4 mL/min at 50 °C. The following gradients were used to wash the metabolites: 0–2 min with solvent A (100%); 2–13 min with solvent B (0–100% in solvent A); 13–15 min with solvent A. In this study, the high-resolution tandem mass spectrometer Xevo G2 XS QTOF (Waters, Milford, USA) was used to analyze the small molecules washed off from the chromatographic column with two different ion modes, precisely positive and negative. In addition, the capillary voltage and cone voltage were operated at 3 kV and 40 V in positive ion modes, and at 1 kV and 40 V in negative ion modes. By using MSE mode for centroid data acquisition, the first level scanning ranges from 50 to 1200 Da with a 0.2 s scanning time. After fragmenting precursor ions according to 20–40 eV of energy, whole debris information was acquired with 0.2 s scanning time. In the process of data collection, the real-time quality correction of LE signal was carried out every 3 s. Generally, to assess the accuracy and stability of the equipment during detection and collection process, the quality-control sample (mixture of all samples) was prepared in advance and tested every 10 samples. Perform data alignment and normalization for the complete data set, composed of multiple analytical blocks, as described in previous report [41].

### Analysis and classification of intracellular metabolites

To investigate the relationship of intracellular metabolites, the raw data of LC–MS/MS were imported into Progenesis QI software (2.2) and analyzed at the default settings parameters. There was a data matrix created with charge, mass-to-charge ratio (m/z) values, peak intensity, retention time and so on. Such a resulting matrix was further filtrated to correct the data by probabilistic quotient normalization and robust LOESS signal correction method based on quality control, which is used to extract information for missing value filling and low-quality ion (relative standard deviation, RSD > 30%). MetaX, the R language analysis package, imported the positive and negative data to analyze fold change and q-value with the t test, the latter was acquired from *p* value by false discovery rate approach. For univariate analysis, principal component analysis (PCA) and partial least square discriminate analysis (PLS-DA) were also used. Meanwhile, the overall contribution of each variable to the PLS-DA model and the importance of the corresponding variable importance in projection (VIP) values were calculated, and those variables of VIP > 1.0 and fold change value > 1.20 or < 0.83 were represented relevant for metabolite classification and the degree of difference and importance in the metabolic analysis. When VIP values > 1 and fold change value > 1.20 or < 0.83, this variable was considered as important metabolite. Metabolites were confirmed by MS/MS scans for the characteristic ions and fragmentation patterns of the compound. The online HMDB database (http://www.hmdb.ca) and KEGG database (http://www.genome.jp/kegg/) were used to align the molecular mass data (m/z) to identify metabo-lites. Commercial reference standards were used to validate and confirm metabolites by comparison of their retention time and MS/MS spectra. We have checked the annotation by using authentic standards (Glucose 6-phosphate, Glycerol, serine, cysteine, SAM, Farnesol, THF-polyglutamate, Amphotericin).

## Supplementary information

**Additional file 1.** Additional figures and tables.

## Abbreviations

AmB: Amphotericin B
AmA: Amphotericin A
DCW: Dry cell weight
HPLC: High performance liquid chromatography
PCA: Principal component analysis
PLS-DA: Partial least square discriminate analysis
VIP: Variable importance in projection
