# Supplementary material for: Amphotericin B biosynthesis in Streptomyces nodosus: quantitative analysis of metabolism via LC–MS/MS based metabolomics for rational design
Source: Microb Cell Fact. 2020 Jan 31;19:18. doi: 10.1186/s12934-020-1290-y (PMC6995120; doi:10.1186/s12934-020-1290-y)
Supplement: Supplementary file 1 — Additional file 1. Additional figures and tables. [file 12934_2020_1290_MOESM1_ESM.docx]

**Additional file**

**Amphotericin B biosynthesis in *Streptomyces nodosus*: quantitative analysis of metabolism via LC-MS/MS based metabolomics for rational design**

Bo Zhang^1,2^, Yi-Teng Zhou1,2, Sheng-Xian Jiang1,2, Yu-Han Zhang1,2, Kai Huang1,2, Zhi-Qiang Liu1,^2*^ and Yu-Guo Zheng^1,2^

^1^*Key Laboratory of Bioorganic Synthesis of Zhejiang Province, College of Biotechnology and Bioengineering, Zhejiang University of Technology, Hangzhou 310014, P. R. China*

^2^*Engineering Research Center of Bioconversion and Bio-purification, Ministry of Education, Zhejiang University of Technology, Hangzhou 310014, P. R. China*

***Correspondence:** Professor Zhi-Qiang Liu, Department, Key Laboratory of Bioorganic Synthesis of Zhejiang Province, College of Biotechnology and Bioengineering, Zhejiang University of Technology, Hangzhou 310014, China

Tel: +86-571-88320614, Fax: +86-571-88320630, E-mail: [microliu@zjut.edu.cn](mailto:microliu@zjut.edu.cn)

**Table S1 Bacterial strains and recombinant plasmids used in this study**

| Strains or plasmids | characteristics | Reference or source |
| --- | --- | --- |
| ***S. nodosus*** |  |  |
| ZJB2016050 | Mutant strain from *Streptomyces* sp. ZJB20130827 amphotericin producer | Zhang et al., 2018  Strain number: CCTCC NO: M  2017426 |
| pJTU1278 | ZJB2016050with pJTU1278 vector | This work |
| amphRI | ZJB2016050with pJTU-EamphRI to overexpress *amphRI* | This work |
| amphRII | ZJB2016050with pJTU-EamphRII to overexpress *amphRII* | This work |
| amphRIV | ZJB2016050with pJTU-EamphRIV tooverexpress *amphRIV* | This work |
| metK | ZJB2016050with pJTU-EmetK tooverexpress *vhb* | This work |
| vhb | ZJB2016050with pJTU-Evhb tooverexpress *vhb* | This work |
| amphG | ZJB2016050with pJTU-EamphG tooverexpress *amphG* | This work |
| amphHG | ZJB2016050with pJTU-EamphHG to overexpress *amphHG* | This work |
| araC | ZJB2016050with pJTU-EaraC tooverexpress *araC* | This work |
| VMR4A | ZJB2016050with pJTU-VMR4AC to overexpress*vhb*, *vhb*, *amphRIV*, *araC* genes | This work |
| VMR4HGA | ZJB2016050with pJTU-VMR4HGA to overexpress*vhb*, *vhb*, *amphRIV*, *araC*, *amphHG* genes | This work |
| ***E.coli*** |  |  |
| DH5α | General cloning host | Vazyme Biotech Co.,Ltd |
| ET12567 (pUZ8002) | Strain for intergeneric conjugation |  |
| **Recombinant plasmids** |  |  |
| pJTU1278 | *E. coli-Streptomyces* conjugative vector |  |
| pJTU-EamphRI | pJTU1278 vector with ermE*p promoter and *amphRI* gene, cluster regulatory gene | This work |
| pJTU-EamphRII | pJTU1278 vector with ermE*p promoter and *amphRII* gene, cluster regulatory gene | This work |
| pJTU-EamphRIV | pJTU1278 vector with ermE*p promoter and *amphRIV* gene, cluster regulatory gene | This work |
| pJTU-Evhb | pJTU1278 vector with ermE*p promoter and *vhb* gene, hyaluronic acid hemoglobin gene | This work |
| pJTU-EmetK | pJTU1278 vector with ermE*p promoter and *metK* gene, S-adenosylmethionine synthetase gene | This work |
| pJTU-EaraC | pJTU1278 vector with ermE*p promoter and *araC* gene, AraC family transcriptional regulator | This work |
| pJTU-EamphG | pJTU1278 vector with ermE*p promoter and *amphG* gene, ABC transporter ATP-binding protein gene | This work |
| pJTU-EamphHG | pJTU1278 vector with ermE*p promoter, *amphH*  and *amphG* gene, both is ABC transporter ATP-binding protein gene | This work |
| pJTU-VM | pJTU1278 vector with ermE*p-vhb-ermE*p-metK | This work |
| pJTU-VMR4A | pJTU1278 vector with erm*E**p-vhb-erm*E**p-metk-  erm*E**p-amphRIV-erm*E**p-araC | This work |
| pJTU-VMR4HGA | pJTU1278 vector with erm*E**p-vhb-erm*E**p-metk-  erm*E**p-amphRIV-erm*E**p-amphHG-erm*E**p-araC | This work |

**Table S2 Primers used in this study**

| **Primer** | **Sequence（5’→3’）** | | **Source** |
| --- | --- | --- | --- |
| ***Recombinant plasmid construction*** | | | |
| *Vhb*-F | CACAGGAGGACCCCAAAGCTTATGCTGGACCAGCAG | | This study |
| *Vhb*-R | GTGCTTGACATTGGGGGATCCTCACTCGACCGCCTG | | This study |
| *MetK*-F | CACAGGAGGACCCCAAAGCTTGTGTCCCGTCGCCTG | | This study |
| *MetK*-R | GTGCTTGACATTGGGGGATCCCTACAGCCCCACTGC | | This study |
| *AmphRI*-F | CACAGGAGGACCCCAAAGCTTGTGATACACGGCCGCGAC | | This study |
| *AmphRI*-R | GTGCTTGACATTGGGGGATCCTCAGGCCCCTTCCGGC | | This study |
| *AmphRIII*-F | CACAGGAGGACCCCAAAGCTTATGCTGCTCGAACGAGG | | This study |
| *AmphRIII*-R | GTGCTTGACATTGGGGGATCCTCAGCCGGGAACGTGCTC | | This study |
| *AmphRIV*-F | CACAGGAGGACCCCAAAGCTTATGCGGAGTACACCCCCA | | This study |
| *AmphRIV*-R | GTGCTTGACATTGGGGGATCCTCAGTCCTTGATGAAGTC | | This study |
| *AmphG*-F | CACAGGAGGACCCCAAAGCTTATGGCGCCGGACGTTCC | | This study |
| *AmphG*-R | GTGCTTGACATTGGGGGATCCTCAGCCGACCGTCACATC | | This study |
| *AmphH*-F | CACAGGAGGACCCCAAAGCTTATGGCCCCGTCGGTGC | | This study |
| *AmphH*-R | GTGCTTGACATTGGGGGATCCTCAGGAACGTCCGGC | | This study |
| *AmphHG*-F | CACAGGAGGACCCCAAAGCTTATGGCCCCGTCGGTGC | | This study |
| *AmphHG*-R | GTGCTTGACATTGGGGGATCCTCAGCCGACCGTCACATC | | This study |
| *AraC*-F | CACAGGAGGACCCCAAAGCTTATGAGCCACGACTCCACC | | This study |
| *AraC*-R | GTGCTTGACATTGGGGGATCCCTACGGTGCGCTGCGCTG | | This study |
| ***Quantitative RT-PCR*** | | | |
| RT(F)-*Vhb* | | CCATCAACATCATCAAGGCC | This study |
| RT(R)-*Vhb* | | CGGCAGGTTCTCGATGTTCT | This study |
| RT(F)-*MetK* | | GGGCCTGATGTTCGGTTATG | This study |
| RT(R)-*MetK* | | GATGGTGACCTGGGTCTTTCC | This study |
| RT(F)-*AmphRIV* | | TCAACTCAGGACGTGAGAAGG | This study |
| RT(R)-*AmphRIV* | | GCCGAACTGCCAGAAGAATC | This study |
| RT(F)-*AraC* | | CTGCCTCCATATCGTGCG | This study |
| RT(R)-*AraC* | | TCTCCACATCGAACTGCTCG | This study |
| RT(F)-*AmphH* | | ACCCGCACCAACGAGGAAC | This study |
| RT(R)-*AmphH* | | CGAAGGCGAGCATCACCAC | This study |
| RT(F)-*AmphG* | | GCATGTCGAGGAGATGTGCG | This study |
| RT(R)-*AmphG* | | AGCCGTTGACGACCTTCCAG | This study |
| RT(F)-16s | | AACGCGAAGAACCTTACCA | This study |
| RT(R)-16s | | AACCCAACATCTCACGACAC | This study |
|  | |  |  |

**Table S3 Identification of different ions**

| Differential ions | | | | | | | Metabolites identification | | | | | |
| --- | --- | --- | --- | --- | --- | --- | --- | --- | --- | --- | --- | --- |
| comparison among groups | Detect mode | R2^d^ | Q2^e^ | Total ion number | Up-regulated number^a^ | Down-regulated number | Identification level 1^b^  ions number | | | Identification level2^c^  ions number | | |
|  |  |  |  |  |  |  | Total | Up | Down | Total | Up | Down |
| group 24h to  group 72h | Positive | 0.9984 | 0.969 | 11145 | 5415 | 5730 | 5556 | 2995 | 2561 | 3253 | 1594 | 1659 |
|  | Negative | 0.981 | 0.7593 | 3068 | 1547 | 1521 | 1077 | 628 | 449 | 628 | 332 | 296 |
| group 72h to  group 120h | Positive | 0.9979 | 0.925 | 4968 | 2465 | 2503 | 2694 | 1198 | 1496 | 1660 | 794 | 866 |
|  | Negative | 0.9794 | 0.7312 | 890 | 391 | 499 | 358 | 162 | 196 | 182 | 78 | 104 |
| group 120h to  group 156h | Positive | 0.9985 | 0.9418 | 3136 | 1572 | 1564 | 1675 | 789 | 886 | 977 | 470 | 507 |
|  | Negative | 0.9984 | 0.9346 | 1893 | 904 | 989 | 716 | 315 | 401 | 404 | 187 | 217 |

1. The number of all differential m/z between two different groups, which including identified ions and unable identified ions
2. The number of identified ions by searching KEGG database associated with primary data (parent ions)
3. The number of identified ions by searching fragmentation information available from KEGG database
4. R2 represents the correlation coefficient of the model, and greater than 0.7 indicates bettercorrelativeability
5. Q2 represents the predictive effect of the PLS-DA model, and greater than 0.5 indicates better predictive ability

**Table S4 Important metabolites identified among different groups and related pathways**

| NO | metabolites | Rt(min) | m/z | Formula | Score | Fragmentation score | VIP | 24 h to 72 h | 72 h to 120 h | 120 h to 156 h | pathway |
| --- | --- | --- | --- | --- | --- | --- | --- | --- | --- | --- | --- |
| 1 | Serine | 8.64 | 106.0508 | C_3_H_7_NO_3_ | 26 | 0 | 1.069609025 | ↓ | —— | —— | Amino acid metabolism |
| 2 | Cysteine | 12.32 | 159.9828 | C_3_H_7_NO_2_S | 37.7 | 3.43 | 1.043753862 | ↓ | —— | —— | Amino acid metabolism |
| 3 | Tyrosine | 2 | 204.0638 | C_9_H_11_NO_3_ | 39.8 | 6.53 | 2.245724547 | ↓ | ↓ | ↑ | Amino acid metabolism |
| 4 | Tryptophan | 4.17 | 227.0803 | C_11_H_12_N_2_O_2_ | 44.6 | 34.5 | 1.320667253 | ↓ | —— | ↑ | Amino acid metabolism |
| 5 | Phenylalanine | 2.04 | 183.1135 | C_9_H_11_NO_2_ | 39.9 | 25.5 | 1.217982646 | ↑ | ↓ | ↑ | Amino acid metabolism |
| 6 | Leucine | 3.91 | 132.103 | C_6_H_13_NO_2_ | 47 | 46.1 | 2.009569834 | —— | ↓ | ↑ | Amino acid metabolism |
| 7 | Valine | 8.65 | 140.069 | C_5_H_11_NO_2_ | 27.5 | 0 | 0.812858467 | ↓ | —— | —— | Amino acid metabolism |
| 8 | Glutamic acid | 4.36 | 130.0507 | C_5_H_9_NO_4_ | 37.7 | 0 | 1.241716778 | ↓ | ↑ | —— | Amino acid metabolism |
| 9 | Histidine | 0.59 | 156.078 | C_6_H_9_N_3_O_2_ | 40.3 | 12 | 2.383589058 | ↓ | ↓ | ↑ | Amino acid metabolism |
| 10 | Proline | 4.22 | 116.0717 | C_5_H_9_NO_2_ | 56.6 | 94.9 | 1.271658459 | —— | ↓ | ↑ | Amino acid metabolism |
| 11 | Arginine | 0.59 | 174.1125 | C_6_H_14_N_4_O_2_ | 39.5 | 4.46 | 1.140338584 | ↑ | ↓ | ↑ | Amino acid metabolism |
| 12 | Glutamine | 3.56 | 147.0771 | C_5_H_10_N_2_O_3_ | 37.6 | 0 | 0.455485721 | —— | —— | —— | Amino acid metabolism |
| 13 | Isoleucine | 3.91 | 132.103 | C_6_H_13_NO_2_ | 47 | 46.1 | 2.009569834 | —— | ↓ | ↑ | Amino acid metabolism |
| 14 | Lysine | 0.53 | 147.1139 | C_6_H_14_N_2_O_2_ | 54.4 | 83.7 | 1.889509062 | ↓ | ↑ | ↓ | Amino acid metabolism |
| 15 | Threonine | 8.96 | 119.059 | C_4_H_9_NO_3_ | 53.9 | 94.7 | 1.530113477 | ↓ | ↑ | —— | Amino acid metabolism |
| 16 | Aspartic acid | 0.59 | 133.0385 | C_4_H_7_NO_4_ | 37.7 | 0 | 1.410133712 | ↓ | ↓ | ↑ | Amino acid metabolism |
| 17 | Methionine | 4.17 | 150.0591 | C_5_H_11_NO_2_S | 36.6 | 0 | 0.85426491 | ↑ | ↓ | ↓ | Amino acid metabolism |
| 18 | Asparagine | 0.67 | 133.0617 | C_4_H_8_N_2_O_3_ | 36 | 0 | 0.815451166 | ↓ | —— | —— | Amino acid metabolism |
| 19 | Methylthioadenosine | 4.25 | 298.0982 | C_11_H_15_N_5_O_3_S | 38.4 | 0 | 2.773090532 | ↓ | ↑ | ↑ | Amino acid metabolism |
| 20 | *S*-Adenosyl-L-methionine(SAM) | 0.65 | 399.1472 | C_15_H_22_N_6_O_5_S | 35.9 | 7.32 | 1.738785589 | ↓ | ↓ | ↓ | Amino acid metabolism |
| 21 | N-Succinyl-2-L-amino-6-oxoheptanedioate | 0.67 | 307.1145 | C_11_H_15_NO_8_ | 42.7 | 20 | 1.720762703 | ↓ | ↑ | —— | Amino acid metabolism |
| 22 | L-Glutamate 5-semialdehyde | 3.63 | 132.0663 | C_5_H_9_NO_3_ | 55 | 82.5 | 1.330392159 | ↓ | ↓ | —— | Amino acid metabolism |
| NO | metabolites | Rt(min) | m/z | Formula | Score | Fragmentation score | VIP | 24 h to 72 h | 72 h to 120 h | 120 h to 156 h | pathway |
| 23 | Homophenylalanine | 4.37 | 180.1031 | C_10_H_13_NO_2_ | 47.9 | 58.4 | 1.206312653 | ↓ | ↑ | ↑ | Amino acid metabolism |
| 25 | Glucose | 0.65 | 203.0542 | C_6_H_12_O_6_ | 38 | 0.637 | 1.684598635 | ↓ | ↓ | ↓ | Sugar metabolism and central metabolic |
| 26 | Glucose 6-phosphate | 0.59 | 260.0306 | C_6_H_13_O_9_P | 39.7 | 5.39 | 1.508877941 | ↓ | ↑ | —— | Sugar metabolism and central metabolic |
| 27 | Fructose 6-phosphate | 0.59 | 260.0306 | C_6_H_13_O_9_P | 40.1 | 7.4 | 1.508877941 | ↓ | ↑ | —— | Sugar metabolism and central metabolic |
| 28 | Glycerol 3-phosphate | 0.59 | 173.0218 | C_3_H_9_O_6_P | 38.5 | 3.31 | 1.421122929 | ↓ | ↓ | ↓ | Sugar metabolism and central metabolic |
| 29 | Sedoheptulose 7-phosphate | 0.59 | 290.0412 | C_7_H_15_O_10_P | 40.5 | 9.44 | 2.284863951 | ↓ | ↑ | ↑ | Sugar metabolism and central metabolic |
| 30 | Mannose | 0.67 | 180.0643 | C_6_H_12_O_6_ | 39.2 | 4.86 | 1.88662279 | ↓ | ↑ | ↓ | Sugar metabolism and central metabolic |
| 31 | N-Acetyl-D-mannosamine | 7.41 | 222.0986 | C_8_H_15_NO_6_ | 43.3 | 34.6 | 1.202342484 | ↓ | ↑ | ↑ | Sugar metabolism and central metabolic |
| 32 | 4-O-beta-D-Glucopyranosyl-D-mannose | 8.4 | 325.1142 | C_12_H_22_O_11_ | 39.5 | 11.5 | 1.795290166 | ↓ | ↓ | —— | Sugar metabolism and central metabolic |
| 33 | Maltose | 8.4 | 325.1142 | C_12_H_22_O_11_ | 39.5 | 11.5 | 1.795290166 | ↓ | ↓ | —— | Sugar metabolism and central metabolic |
| 34 | 3-Propylmalate | 5.22 | 159.0661 | C_7_H_12_O_5_ | 54.2 | 86.2 | 1.572287782 | ↓ | ↑ | ↑ | Sugar metabolism and central metabolic |
| 35 | Acetylpyruvate | 0.67 | 113.0233 | C_5_H_6_O_4_ | 38.1 | 4.34 | 1.530557299 | —— | ↓ | —— | Sugar metabolism and central metabolic |
| 36 | Ribulose | 3.38 | 133.0505 | C_5_H_10_O_5_ | 49.8 | 83.1 | 1.054220577 | ↓ | —— | —— | Sugar metabolism and central metabolic |
| 37 | Fumarate | 4.1 | 134.0447 | C_4_H_4_O_4_ | 38.8 | 0 | 0.776490877 | —— | ↓ | —— | Sugar metabolism and central metabolic |
| 38 | Galactose | 12.84 | 203.0537 | C_6_H_12_O_6_ | 37.2 | 0 | 1.037306001 | ↓ | —— | —— | Sugar metabolism and central metabolic |
| 39 | Hexadecanoic acid | 9.25 | 257.2486 | C_16_H_32_O_2_ | 43.4 | 25.4 | 1.953670884 | ↓ | ↓ | ↑ | Fatty acid biosynthesis |
| 40 | Tetradecanoic acid | 8.8 | 211.2059 | C_14_H_28_O_2_ | 37.6 | 0 | 1.527353718 | ↓ | ↑ | ↓ | Fatty acid biosynthesis |
| 41 | Icosenoic acid | 9.68 | 328.3227 | C_20_H_38_O_2_ | 36.8 | 0.726 | 1.308094543 | ↓ | —— | ↓ | Fatty acid biosynthesis |
| 42 | Octadecanoic acid | 8.91 | 267.2687 | C_18_H_36_O_2_ | 36.4 | 0 | 1.16948913 | ↓ | —— | —— | Fatty acid biosynthesis |
| 43 | Decanoic acid | 8.25 | 211.1108 | C_10_H_20_O_2_ | 40.3 | 11.7 | 1.010717142 | ↓ | —— | —— | Fatty acid biosynthesis |
| 44 | Dodecanoic acid | 5.56 | 218.2127 | C_12_H_24_O_2_ | 38.2 | 0 | 1.072177732 | ↓ | ↑ | —— | Fatty acid biosynthesis |
| 45 | Icosatrienoic acid | 9.33 | 324.2913 | C_20_H_34_O_2_ | 37.1 | 5.56 | 1.025906942 | ↓ | ↑ | —— | Fatty acid biosynthesis |
| 46 | Hexadecanal | 7.82 | 258.2802 | C_16_H_32_O | 36.9 | 0 | 1.183875968 | ↓ | —— | —— | Fatty acid biosynthesis |
| 47 | Farnesol | 7.57 | 222.1994 | C_15_H_26_O | 38.6 | 0 | 3.452114499 | ↓ | ↑ | ↑ | Terpenoid backbone biosynthesis |
| 48 | Farnesyl diphosphate | 0.63 | 405.1189 | C_15_H_28_O_7_P_2_ | 37.9 | 3.86 | 3.125210737 | ↓ | ↓ | ↑ | Terpenoid backbone biosynthesis |
| 49 | Dihydrogeranylgeranyl diphosphate | 3.41 | 453.213 | C_20_H_38_O_7_P_2_ | 36 | 0.51 | 2.161518484 | ↑ | ↓ | —— | Terpenoid backbone biosynthesis |
| 50 | Heptaprenyl diphosphate; | 6.81 | 677.3712 | C_35_H_60_O_7_P_2_ | 42.6 | 31.9 | 1.601998914 | ↑ | —— | —— | Terpenoid backbone biosynthesis |
| NO | metabolites | Rt(min) | m/z | Formula | Score | Fragmentation score | VIP | 24 h to 72 h | 72 h to 120 h | 120 h to 156 h | pathway |
| 51 | Hexaprenyl diphosphate; | 7.16 | 609.3046 | C_30_H_52_O_7_P_2_ | 38.3 | 4.72 | 1.383436903 | ↑ | ↓ | ↓ | Terpenoid backbone biosynthesis |
| 52 | Dolichyl diphosphate | 6.81 | 677.3712 | C_20_H_38_O_7_P_2_ | 19.6 | 0 | 1.601998914 | ↑ | —— | —— | Terpenoid backbone biosynthesis |
| 53 | Presqualene diphosphate | 6.98 | 609.3052 | C_30_H_52_O_7_P_2_ | 37.1 | 0 | 1.507880323 | ↑ | ↓ | ↓ | Terpenoid backbone biosynthesis |
| 54 | Octaprenyl diphosphate | 4.33 | 761.412 | C_40_H_68_O_7_P_2_ | 36.3 | 4.45 | 1.379204629 | ↑ | ↓ | ↓ | Terpenoid backbone biosynthesis |
| 55 | Geranylgeranyl diphosphate | 4.91 | 450.1971 | C_20_H_36_O_7_P_2_ | 35 | 0 | 1.148377076 | ↑ | —— | —— | Terpenoid backbone biosynthesis |
| 56 | phytyl diphosphate | 10.12 | 439.2401 | C_20_H_42_O_7_P_2_ | 35.8 | 0 | 1.128273301 | ↓ | —— | —— | Terpenoid backbone biosynthesis |
| 57 | mevalonate | 0.67 | 166.1076 | C_6_H_12_O_4_ | 35.8 | 6.29 | 1.012482462 | —— | ↓ | —— | Terpenoid backbone biosynthesis |
| 58 | Uric acid ribonucleoside | 0.57 | 318.1047 | C_10_H_12_N_4_O_7_ | 38.8 | 2.8 | 2.832480739 | ↓ | ↓ | ↑ | Purine and pyrimidine metabolism |
| 59 | 3',5'-Cyclic CMP | 0.59 | 344.0061 | C_9_H_12_N_3_O_7_P | 38.6 | 1.89 | 2.45693254 | ↓ | ↑ | ↓ | Purine and pyrimidine metabolism |
| 60 | Kinetin | 2.05 | 216.0881 | C_10_H_9_N_5_O | 40.5 | 6.64 | 1.927985277 | ↑ | ↑ | ↓ | Purine and pyrimidine metabolism |
| 61 | XMP(Xanthylic acid; XMP) | 0.59 | 403.007 | C_10_H_13_N_4_O_9_P | 36.9 | 1.48 | 1.889616275 | ↓ | ↑ | ↓ | Purine and pyrimidine metabolism |
| 62 | N7-Methylguanosine | 3.96 | 281.1143 | C_11_H_16_N_5_O_5_ | 38.2 | 3.83 | 1.630550172 | ↑ | ↑ | ↓ | Purine and pyrimidine metabolism |
| 63 | CMP | 0.67 | 324.0596 | C_9_H_14_N_3_O_8_P | 38.5 | 1.86 | 1.565382788 | ↓ | ↑ | —— | Purine and pyrimidine metabolism |
| 64 | 5-Amino-4-imidazole carboxylate | 1.51 | 110.0358 | C_4_H_5_N_3_O_2_ | 56.4 | 94.1 | 1.547788997 | ↓ | ↑ | —— | Purine and pyrimidine metabolism |
| 65 | 5-Ureido-4-imidazole carboxylate | 0.67 | 153.04 | C_5_H_6_N_4_O_3_ | 41.7 | 16.3 | 1.439705751 | ↓ | ↑ | —— | Purine and pyrimidine metabolism |
| 66 | Uridine | 0.66 | 244.0707 | C_9_H_12_N_2_O_6_ | 43.9 | 31.1 | 1.418696559 | ↓ | ↓ | ↑ | Purine and pyrimidine metabolism |
| 67 | Cytidine | 0.62 | 261.1205 | C_9_H_13_N_3_O_5_ | 38.8 | 5.01 | 1.358579765 | ↓ | ↓ | ↑ | Purine and pyrimidine metabolism |
| 68 | Hypoxanthine | 1.51 | 136.0384 | C_5_H_4_N_4_O | 54.3 | 84.1 | 1.279542142 | ↓ | ↑ | —— | Purine and pyrimidine metabolism |
| 69 | dGMP | 4.45 | 348.0715 | C_10_H_14_N_5_O_7_P | 37.7 | 0 | 1.265420066 | ↓ | ↑ | ↓ | Purine and pyrimidine metabolism |
| 70 | Inosine | 3.84 | 251.0784 | C_10_H_12_N_4_O_5_ | 38.9 | 0.686 | 1.195611968 | ↓ | ↑ | —— | Purine and pyrimidine metabolism |
| 71 | O6-Methyl-2'-deoxyguanosine | 6.96 | 299.1486 | C_11_H_15_N_5_O_4_ | 40 | 11.3 | 1.300884423 | ↑ | ↑ | —— | Purine and pyrimidine metabolism |
| 72 | dADP | 0.57 | 394.0302 | C_10_H_15_N_5_O_9_P_2_ | 38.6 | 0 | 0.937601855 | ↑ | ↑ | ↓ | Purine and pyrimidine metabolism |
| 73 | deoxyguanosine | 3.48 | 267.0983 | C_10_H_13_N_5_O_4_ | 49.6 | 55.1 | 0.632019641 | ↓ | —— | —— | Purine and pyrimidine metabolism |
| 74 | Deoxyadenosine | 0.65 | 252.1088 | C_10_H_13_N_5_O_3_ | 46.2 | 35.7 | 0.457898515 | —— | —— | —— | Purine and pyrimidine metabolism |
| 75 | Deoxyinosine | 3.5 | 252.0873 | C_10_H_12_N_4_O_4_ | 41.3 | 15.4 | 0.895151345 | —— | —— | —— | Purine and pyrimidine metabolism |
| 76 | Adenine | 1.14 | 136.0628 | C_5_H_5_N_5_ | 39.7 | 14 | 0.922216258 | ↓ | ↑ | ↓ | Purine and pyrimidine metabolism |
| 77 | Guanine | 3.48 | 134.0472 | C_5_H_5_N_5_O | 52.2 | 76.1 | 0.823977411 | ↓ | ↓ | —— | Purine and pyrimidine metabolism |
| NO | metabolites | Rt(min) | m/z | Formula | Score | Fragmentation score | VIP | 24 h to 72 h | 72 h to 120 h | 120 h to 156 h | pathway |
| 78 | Thymine | 3.75 | 126.0438 | C_5_H_6_N_2_O_2_ | 45.3 | 35.2 | 0.761443076 | ↓ | ↓ | —— | Purine and pyrimidine metabolism |
| 79 | 7-Carboxy-7-carbaguanine | 5.04 | 195.0516 | C_7_H_6_N_4_O_3_ | 37.8 | 0 | 3.044060305 | ↓ | ↑ | ↓ | Folate biosynthesis and one carbon pool |
| 80 | THF-polyglutamate | 8.65 | 686.252 | C_24_H_30_N_8_O_9_ | 19.7 | 0 | 2.890574863 | ↓ | ↓ | ↓ | Folate biosynthesis and one carbon pool |
| 81 | 5,10-Methylenetetrahydrofolate | 3.87 | 440.1687 | C_20_H_23_N_7_O_6_ | 41 | 16.6 | 2.488054755 | ↓ | —— | ↑ | Folate biosynthesis and one carbon pool |
| 82 | 7,8-Dihydrofolate | 3.83 | 426.1521 | C_19_H_21_N_7_O_6_ | 34.8 | 0.275 | 2.42975247 | —— | —— | ↑ | Folate biosynthesis and one carbon pool |
| 83 | 5-Formiminotetrahydrofolate | 3.42 | 490.2161 | C_20_H_24_N_8_O_6_ | 48.6 | 52.4 | 1.909647401 | ↓ | ↓ | ↑ | Folate biosynthesis and one carbon pool |
| 84 | 5,10-Methenyltetrahydrofolate | 4.48 | 457.1712 | C_20_H_22_N_7_O_6_ | 39.1 | 10 | 1.58192323 | ↓ | ↑ | ↑ | Folate biosynthesis and one carbon pool |
| 85 | 5,6,7,8-Tetrahydrofolate | 7.05 | 463.2058 | C_19_H_23_N_7_O_6_ | 44.5 | 32.2 | 1.076451372 | ↑ | ↑ | —— | Folate biosynthesis and one carbon pool |
| 86 | Shikimate | 3.38 | 157.0503 | C_7_H_10_O_5_ | 48.9 | 54.1 | 1.191572627 | —— | ↑ | —— | Folate biosynthesis and one carbon pool |
| 87 | Convallamarogenin | 6.05 | 453.2969 | C_27_H_42_O_4_ | 57.4 | 97.1 | 5.490355492 | ↓ | —— | ↓ | Biosynthesis of secondary metabolites |
| 88 | Gentamicin | 6.56 | 481.3374 | C_20_H_41_N_5_O_7_ | 37.9 | 10.8 | 4.615476315 | ↓ | —— | ↓ | Biosynthesis of secondary metabolites |
| 89 | Bluensomycin | 7.75 | 586.2579 | C_21_H_39_N_5_O_14_ | 35.5 | 2.08 | 2.586323019 | ↑ | ↓ | ↓ | Biosynthesis of secondary metabolites |
| 90 | Amphotericin B | 4.52 | 946.4727 | C_47_H_73_NO_17_ | 49.3 | 61.9 | 2.280512756 | ↑ | ↑ | —— | Biosynthesis of secondary metabolites |
| 91 | Avermectin | 7.66 | 715.4085 | C_40_H_60_O_12_ | 43.8 | 30.1 | 2.273911441 | ↑ | ↑ | —— | Biosynthesis of secondary metabolites |
| 92 | Tylosin | 7.3 | 789.4707 | C_39_H_65_NO_14_ | 53.1 | 75.2 | 2.703048098 | —— | ↑ | ↑ | Biosynthesis of secondary metabolites |
| 93 | Ectoine | 3.65 | 143.0821 | C_6_H_10_N_2_O_2_ | 42.3 | 23.6 | 2.041230183 | —— | —— | ↑ | Biosynthesis of secondary metabolites |
| 94 | Urdamycinone F | 0.69 | 487.161 | C_25_H_28_O_11_ | 39.2 | 6.53 | 1.674191166 | ↓ | ↓ | ↓ | Biosynthesis of secondary metabolites |
| 95 | delta-n-Amylbutyrolactone | 6.22 | 174.1497 | C_9_H_16_O_2_ | 36.8 | 0 | 1.256296198 | ↓ | —— | —— | Biosynthesis of secondary metabolites |
| 96 | Albaflavenone | 6.15 | 219.1755 | C_15_H_22_O | 50.6 | 62.6 | 1.235048343 | ↓ | —— | —— | Biosynthesis of secondary metabolites |
| 97 | Geosmin | 9.31 | 165.1646 | C_12_H_22_O | 50.8 | 61.8 | 1.005125749 | ↓ | —— | —— | Biosynthesis of secondary metabolites |
| 98 | Aerobactin | 4.05 | 582.2661 | C_22_H_36_N_4_O_13_ | 36.3 | 0 | 0.907632052 | ↑ | —— | ↑ | Biosynthesis of secondary metabolites |
| 99 | 5-Hydroxyectoine | 3.75 | 141.0667 | C_6_H_10_N_2_O_3_ | 52.3 | 69.8 | 0.79269423 | —— | —— | ↓ | Biosynthesis of secondary metabolites |
| 100 | Coelichelin | 7.75 | 583.307 | C_21_H_39_N_7_O_11_ | 34.2 | 1.89 | 0.634346736 | ↑ | —— | —— | Biosynthesis of secondary metabolites |


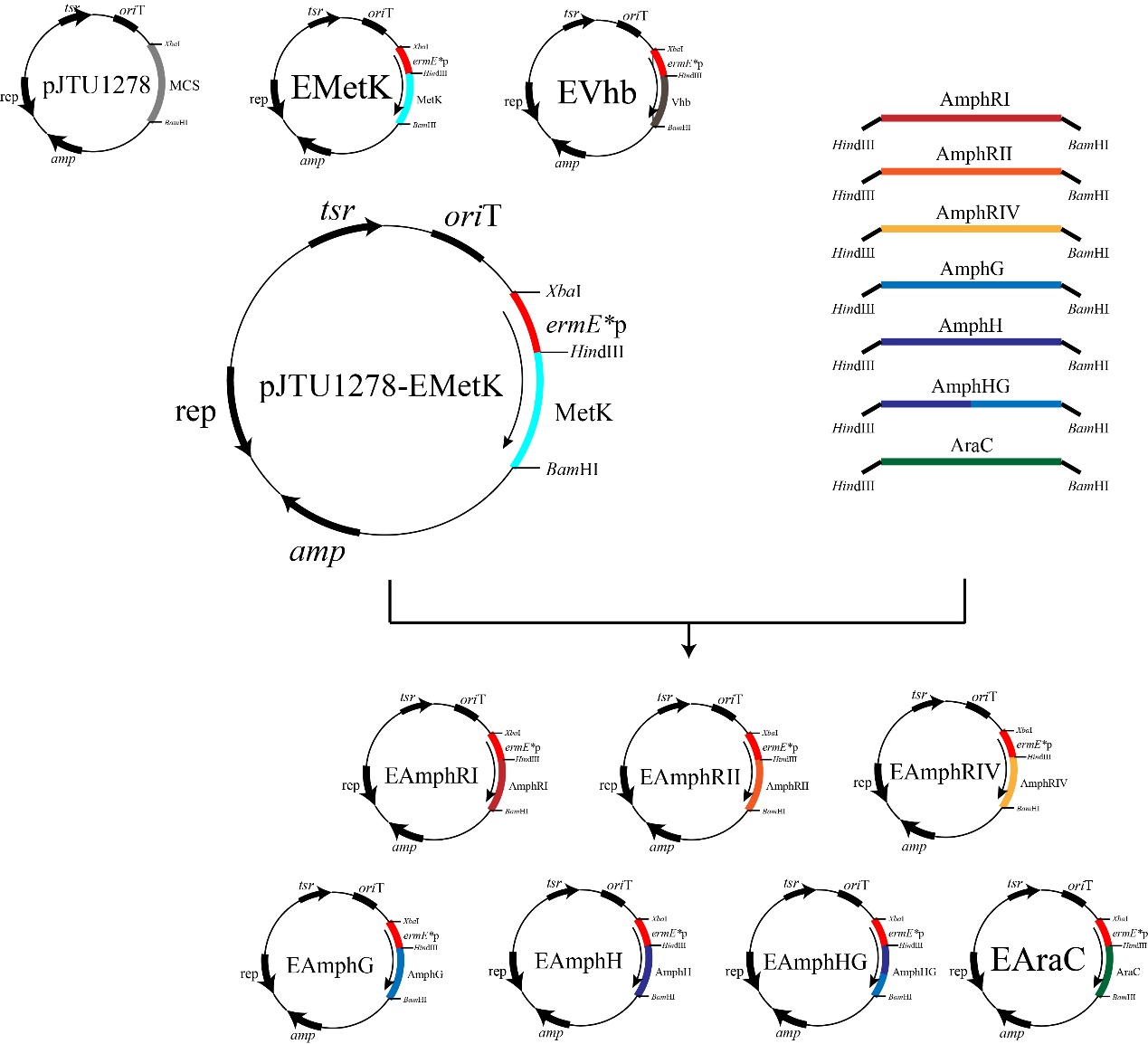


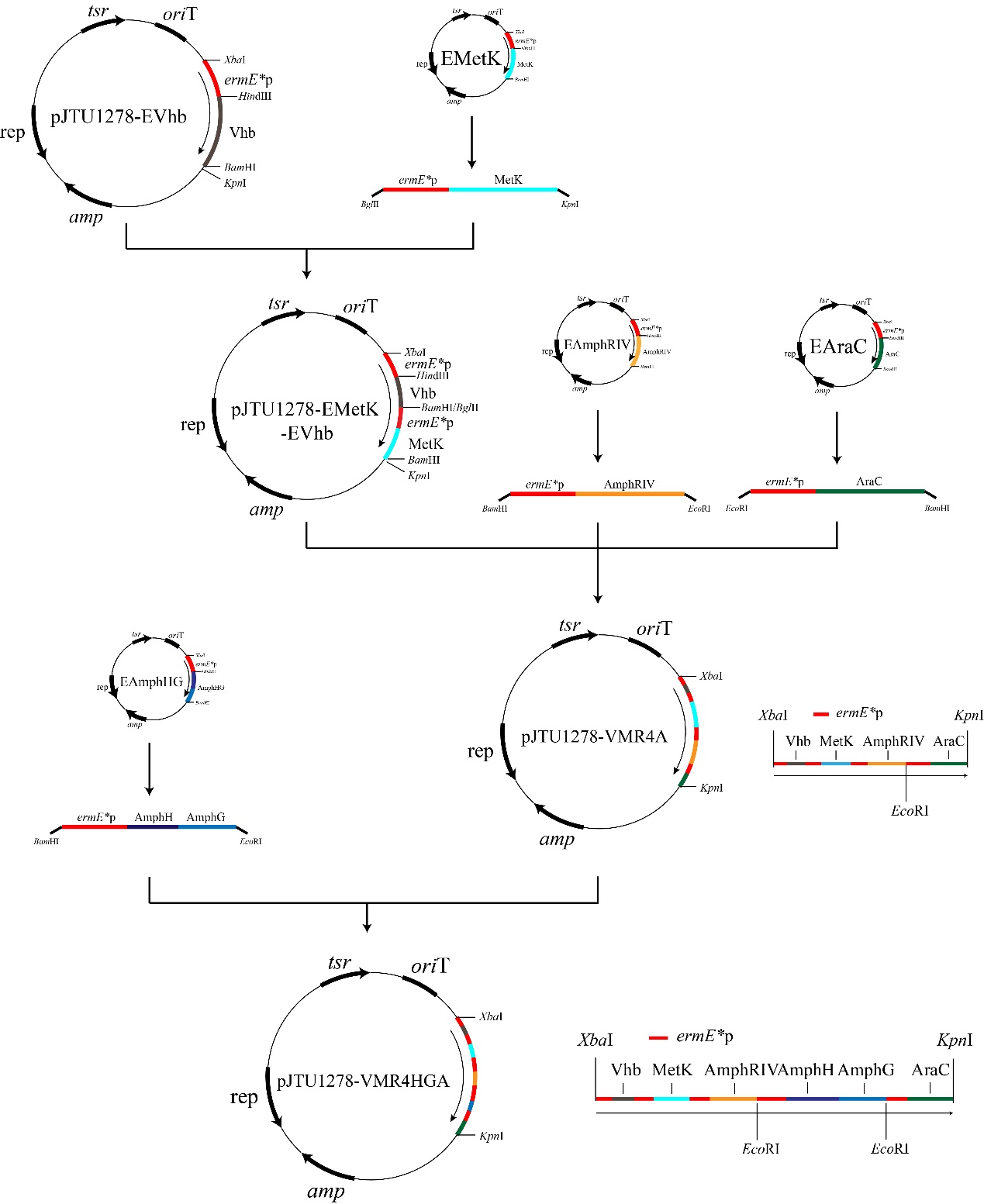


**Fig. S1 Structures of plasmids used in this study.**

Detailed description on constructions of these plasmids is shown in Section 2 Construction of Plasmids.





**Fig. S2 RT-qPCR analysis of transcriptional levels of single gene and multi-genes expression in *S. nodosus* ZJB2016050.**

All experiments were performed in triplicate; error bars denote standard deviation of the mean.





**Fig. S3 The part of pathway enrichment in top with the p-value＜0.05.**

Black and grey bars indicate the identified differential metabolites and total metabolites in the metabolic pathway, respectively.


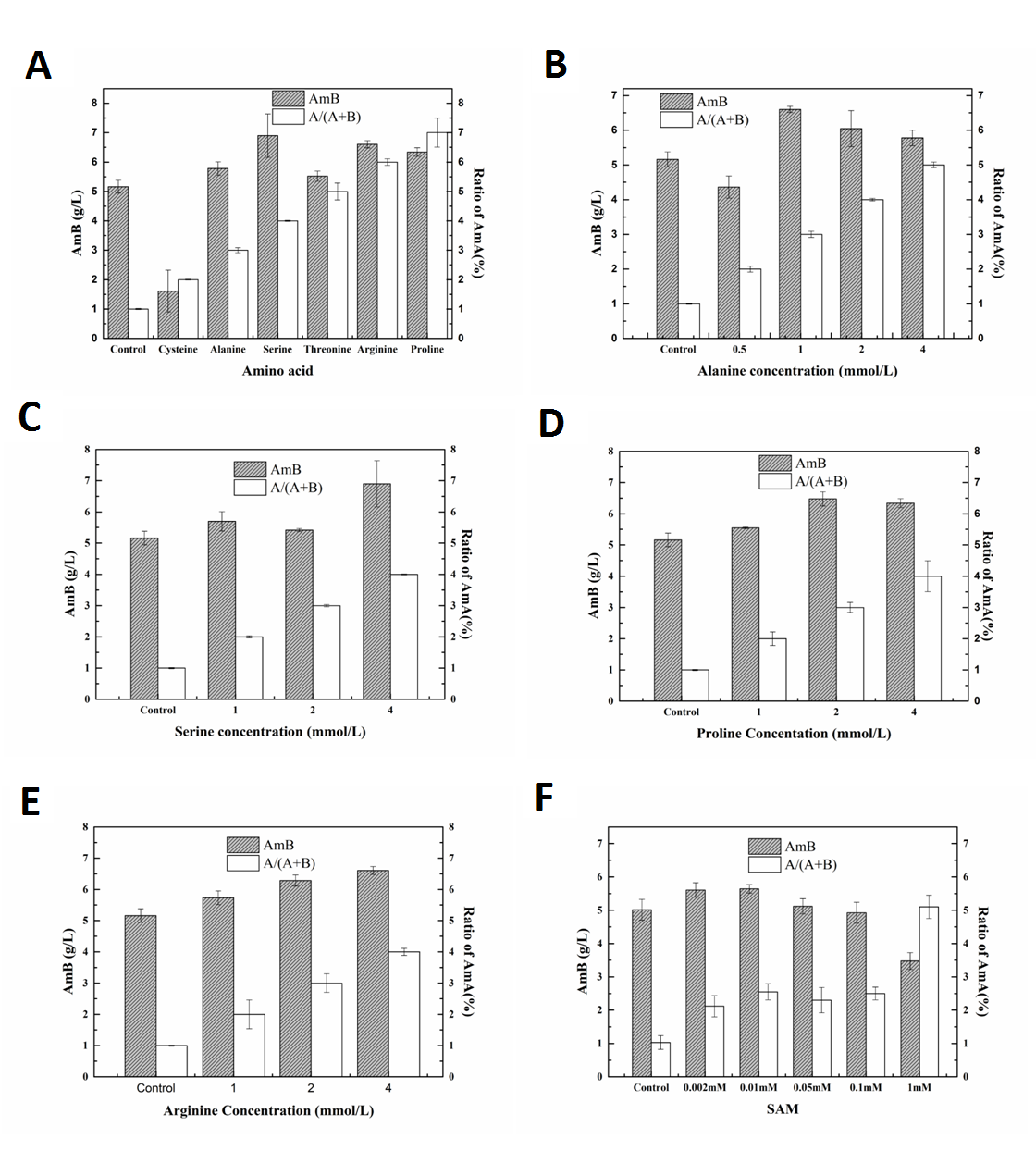


**Fig. S4 The production of AmB for different metabolites addition with strain *S. nodosus* ZJB2016050.**

(A) Effects of amino acids addition (1 mM) on AmB production. (B) Effects of different alanine concentration on AmB production. (C) Effects of different serine concentration on AmB production. (D) Effects of different proline concentration on AmB production. (E) Effects of different arginine concentration on AmB production. (F) Effects of different SAM concentration on AmB production. Each value is a mean of three experiments. Error bars show standard derivation among three experiments.


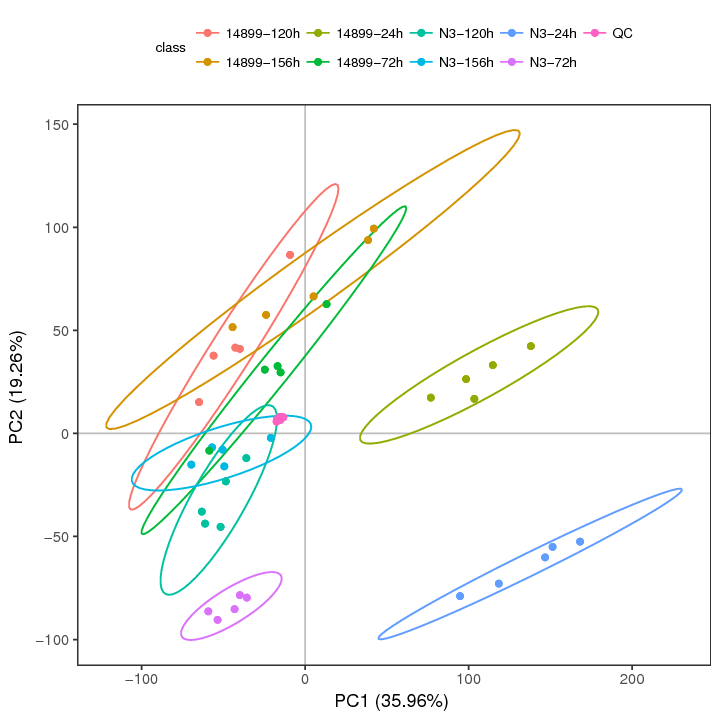


**Fig. S5 PCA plots including all QC samples.**
